# Supplementary material for: Identification of photoperiod-induced specific miRNAs in the adrenal glands of Sunite sheep (Ovis aries)
Source: Front Vet Sci. 2022 Jul 22;9:888207. doi: 10.3389/fvets.2022.888207 (PMC9354845; doi:10.3389/fvets.2022.888207)
Supplement: Supplementary file 1 [file Data_Sheet_1.ZIP › Supplementary file/miRDeep2 file.zip!/miRDeep2 file/Know_Structure/LP42A2/oar-mir-376a.pdf]

miRBase precursor : oar-mir-376a
Total read count : 178
oar-miR-376a-5p read count : 111
oar-miR-376a-3p read count : 62
remaining reads : 5

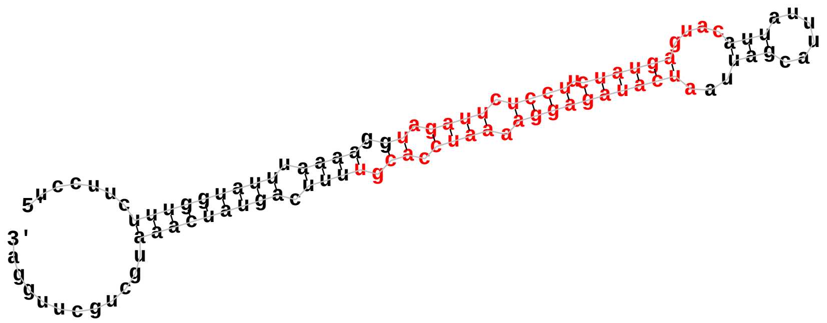

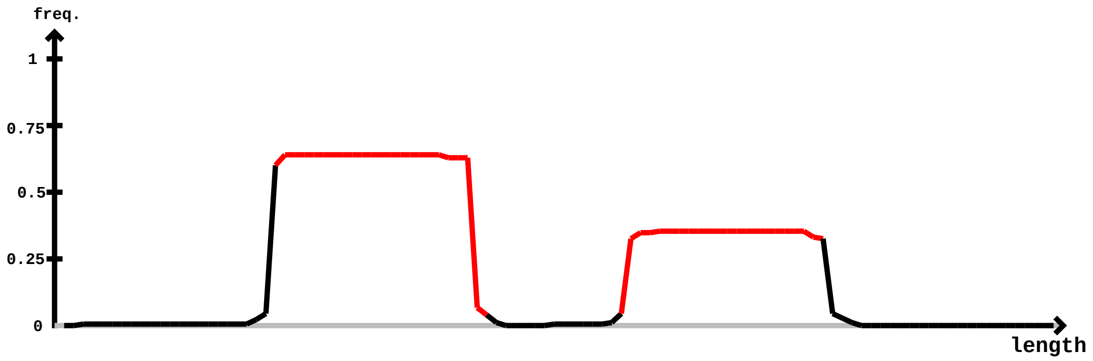

oar-miR-376a-5p

oar-miR-376a-3p

```
5'- uccuucuuugguauuuaaaagguagauucuccuucuaugaguacauuauuuacgauuaaucauagaggaaaauccacguuuucaguaucaaaugcugcuugga -3'   exp
    ......((((((((.((((.((.((((.((((.((((((....(((......)))...)))))))))).)))).)).)))).))))))))............. reads  mm  sample
    .ccuucuuugguauuuaaaag.................................................................................. 1      0   seq
    ...................agguagauucuccuucuaugagu............................................................. 3      0   seq
    ....................gguagauucuccuucuaugagu............................................................. 4      0   seq
    .....................guagauucuccuucuaug................................................................ 2      0   seq
    .....................guagauucuccuucuaugagu............................................................. 89     0   seq
    .....................guagauucuccuucuaugagua............................................................ 4      0   seq
    .....................guagauucuccuucuaugaguac........................................................... 5      0   seq
    ......................uagauucuccuucuaugagu............................................................. 4      0   seq
    ......................uagauucuccuucuaugagua............................................................ 1      0   seq
    ......................uagauucuccuucuaugaguaca.......................................................... 2      0   seq
    ..................................................uacgauuaaucauagaggaaaauccacgu........................ 1      0   seq
    ........................................................uaaucauagaggaaaauccac.......................... 1      0   seq
    .........................................................aaucauagaggaaaauccacgu........................ 5      0   seq
    .........................................................aaucauagaggaaaauccacguu....................... 1      0   seq
    ..........................................................aucauagaggaaaauccac.......................... 3      0   seq
    ..........................................................aucauagaggaaaauccacg......................... 1      0   seq
    ..........................................................aucauagaggaaaauccacgu........................ 44     0   seq
    ..........................................................aucauagaggaaaauccacguu....................... 2      0   seq
    ...........................................................ucauagaggaaaauccacguuu...................... 3      0   seq
    ...........................................................ucauagaggaaaauccacguuuu..................... 1      0   seq
    .............................................................auagaggaaaauccacguuuu..................... 1      0   seq
```
